# High Photoresponsivity Ge-dot PhotoMOSFETs for Low-power Monolithically-Integrated Si Optical Interconnects

**DOI:** 10.1038/srep44402

**Published:** 2017-03-16

**Authors:** Ming-Hao Kuo, Meng-Chun Lee, Horng-Chih Lin, Tom George, Pei-Wen Li

**Affiliations:** 1 Department of Electrical Engineering, National Central University, ZhongLi, Taiwan, R.O.C; 2 Department of Electronics Engineering, National Chiao Tung University, HsinChu, Taiwan, R.O.C

## Abstract

We report the demonstration of high-photoresponsivity Ge-dot photoMOSFETs in a standard MOS configuration for the detection of 850–1550 nm illumination. Each device has a self-organized, gate-stacking heterostructure of SiO_2_/Ge-dot/SiO_2_/SiGe-channel which is simultaneously fabricated in a single oxidation step. Superior control of the geometrical size and chemical composition for our Ge nanodots/SiO_2_/Si_1-*x*_Ge_*x*_-shell MOS structure enables the practically-achievable, gate-stacking design for our Ge-dot photoMOSFETs. Both the gate oxide thickness and the diameter of the Ge dots are controllable. Large photocurrent enhancement was achieved for our Ge-dot photoMOSFETs when electrically-biased at ON- and OFF-states based on the Ge dot mediating photovoltaic and photoconductive effects, respectively. Both photoelectric conversion efficiency and response speed are significantly improved by reducing the gate-oxide thickness from 38.5 nm to 3.5 nm, and by decreasing Ge-dot size from 90 nm to 50 nm for a given areal density of Ge dots. Photoresponsivity (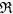) values as high as 1.2 × 10^4^ A/W and 300 A/W are measured for 10 nW illumination at 850 nm and 1550 nm, respectively. A response time of 0.48 ns and a 3 dB-frequency of 2 GHz were achieved for 50 nm-Ge-dot photoMOSFETs with channel lengths of 3 μm under pulsed 850 nm illumination.

The Moore’s law juggernaut may finally be slowing down because of severe short channel effects experienced by active transistors, and also due to formidable interconnect bottlenecks arising from the current copper-trace based technology. Electrical interconnect performance has degraded significantly in terms of both speed and power dissipation in large part due to the fact that aggressive downscaling in feature sizes negative impacts both the resistances and inductances of metal wires. Si optical interconnects have become an increasingly attractive alternative since they promise to provide greater bandwidth, lower power consumption, decreased latency, higher resistance to electromagnetic interference, and reduced signal crosstalk^1^. However, optical interconnects can only hope to replace electrical interconnects when these have both better performance and can be cost-effectively manufactured in high volumes.

The major challenge for the adoption of silicon optical interconnects lies in achieving seamless, monolithic integration of photonics and electronics within standard CMOS processing technology. Among the various material choices for Si photonics, Ge is particularly attractive thanks to its pseudo-direct bandgap structure and compatibility with Si integrated-circuit technology^2^. The effectiveness and functionality of many important key components for Si/Ge photonics, including photodetectors^3^, modulators^4^, and light sources^5^, have already been demonstrated. Given their micrometer-scales in thickness and lateral dimensions, however, these Ge/Si heterostructures are either too thick or too large to be directly integrated with the prevailing submicron or even nanometer-scale Si electronic devices. This size incompatibility, in particular, affects the receiver component which requires high-responsivity, high-speed, and ultra-low capacitance photodiodes to be integrated in close proximity to the transimpedance and limiting amplifiers thereby leading to substantial penalties in power dissipation, device area, latency, and noise.

Using a phototransistor (PT) that is a photodetector with internal gain may help mitigate these issues. In fact, several attempts have been made in the design and fabrication of phototransistors with different device configurations including MOSFETs^6^,^7^, JFETs^8^,^9^, and BJTs^10^,^11^,^12^. A photoMOSFET in a standard MOS configuration that eliminates metal wires connecting photodiodes and their proximal MOSFETs in receiver circuits is the most promising solution to simultaneously realize photodetection and electrical amplification within a single device.

Recently, we have demonstrated a unique, self-organized, gate-stacking MOS heterostructure of SiO_2_/Ge nanodots/SiO_2_/Si_1-x_Ge_x_-shell over the Si substrate. The key novelty of our MOS gate-stacking structure lies in its simplicity and elegance of being “instantaneously” produced within a single oxidation step. Our approach effectively eliminates complicated surface cleaning and passivation processes that are quite difficult to achieve for Ge-based MOS gate-stacking structures produced using conventional fabrication processes. Superior control over the geometrical size and chemical composition of our Ge nanodot/SiO_2_/Si_1-x_Ge_x_-shell MOS structure is achieved as evidenced by our ability to produce size-tunable^13^, 5–100 nm-diameter Ge nanodots, 3–40 nm-thick SiO_2_ gate oxide, and 2–22 nm-thick Si_1-*x*_Ge_*x*_ channels with *x* = 0.5–0.8. Our achievement indeed paves the way for realizing a practically-achievable, core building block for next-generation, Ge-based MOS nanoelectronic and nanophotonic devices. In this paper, we further advance the gate-stacking design of our Ge photoMOSFET in order to boost its performance in terms of reduced dark current, and increased photoresponsivity, quantum efficiency, and response speed by tailoring the Ge-dot diameter and the gate oxide thickness appropriately.

## Results

### Ge-dot PhotoMOSFETs structure

**Figure 1:**
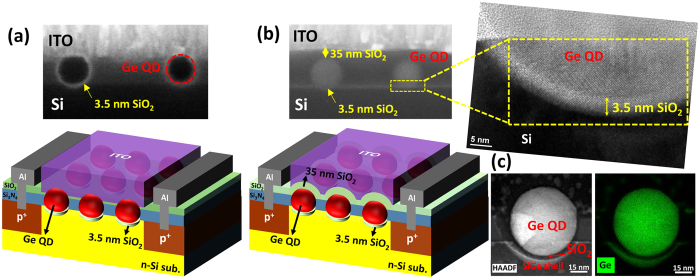
Ge-dot phototransistors (PTs) have a self-organized, gate-stacking heterostructure of a SiO_2_/Ge-dot/SiO_2_/SiGe-channel, which is simultaneously produced in a single oxidation step. Cross-sectional scanning electron microscopic micrographs and 3D schematic diagrams of Ge-dot PTs with (**a**) *t*_ox_ = 3.5 nm and (**b**) *t*_ox_ = 38.5 nm showing our ability to precisely control the sizes of the Ge nanodots and the thicknesses of the SiO_2_ gate oxide. Notably, there exists a 3.5 nm-thick interfacial layer of SiO_2_ surrounding the Ge dots as a result of an exquisitely-controlled dynamic balance between the fluxes of oxygen and silicon interstitials. (**c**) EDX elemental *x*-ray mapping micrographs of a SiO_2_/Ge-QD/SiO_2_/SiGe-shell heterostructure over the Si substrate.

Schematic diagrams and cross-sectional transmission electron micrographs (CTEM) of Ge-dot MOS-PTs are shown in Fig. 1. The Ge-dot PTs have a self-aligned, gate-stacking heterostructure of top SiO_2_/Ge-dot/interfacial SiO_2_/SiGe-channel which is simultaneously produced in a single oxidation step of Si_0.85_Ge_0.15_ nano-pillars fabricated over a buffer layer of Si_3_N_4_ on top of the *n*-Si substrate. HRSTEM micrograph and EDX mapping micrograph examinations confirm the high chemical purity of each Ge QD, as shown in Fig. 1(c). Two thicknesses of 0 nm and 35 nm for the top oxide layer above the Ge dots are produced by a direct etch-back process, as shown in Fig. 1(a,b), respectively. It is interesting to note that there exists a 3.5 nm-thick interfacial layer of SiO_2_ surrounding the Ge dots as a result of an exquisitely-controlled dynamic balance between the fluxes of oxygen and silicon interstitials^14^. In effect, this leads to two gate-oxide thicknesses (***t***_*ox*_) of 3.5 nm and 38.5 nm for the Ge-dot gate stacks.

### Photocurrent characterization

**Figure 2:**
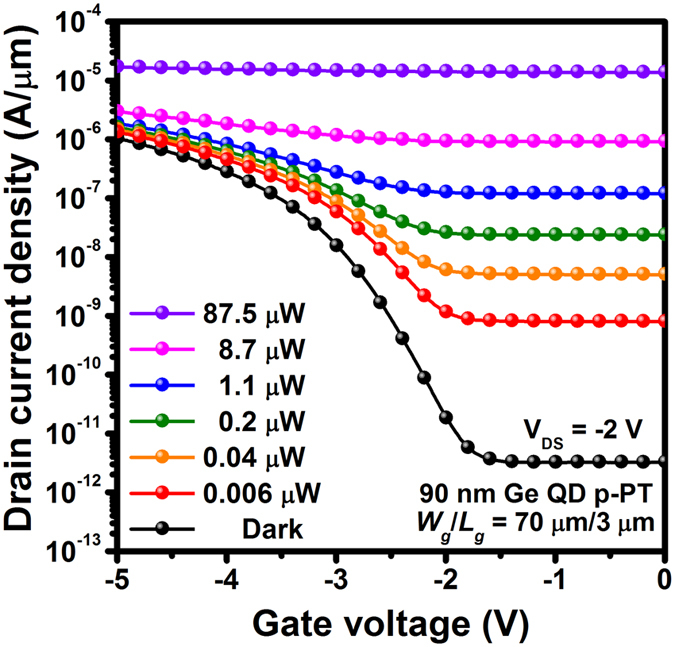
*I*_D_-*V*_G_ characteristics of 90 nm Ge-dot *p*-PTs (*W*_g_/*L*_g_ = 70 μm/3 μm) with *t*_ox_ = 38.5 nm under variable-power 850 nm illumination from 0.006–87.5 μW. Large photocurrent enhancement was achieved for our Ge-dot PTs when electrically-biased in the ON- and OFF-states based on the Ge dot mediating photovoltaic and photoconductive effects, respectively.

Figure 2 shows *I*_D_-*V*_G_ characteristics of 90 nm-diameter Ge-dot p-MOSFETs biased at *V*_*D*_ = −2 V and measured either under darkness or variable-incident power (*P*_IN_) 850 nm illumination. Ge-dot *p*-MOSFETs show typical transfer curves with ON-OFF current ratios (*I*_ON_*/I*_OFF_) of as high as 10^6^ measured under darkness. One can clearly see that 850 nm illumination does indeed increase drain current for the Ge-dot p-PTs across the entire experimental gate voltage range. For example, optical pumping at *P*_IN_ = 87.5μW significantly increases *I*_ON_ and *I*_OFF_ for Ge-dot *p*-PTs by a factor of 20 and 8 × 10^6^ in magnitude, respectively, in comparison to the corresponding dark current values. This indicates that a high-level injection of photocarriers generates very high photocurrents that surpass thermionic and tunneling currents for both the ON- and OFF-states. In contrast, there appears to be only a relatively small enhancement in *I*_OFF_ (at most 10x) and also no visible deviation in *I*_ON_ for the control Si-MOSFET (i.e., not containing Ge dots) under similar variable illumination conditions (not shown in Fig. 2).

**Figure 3:**
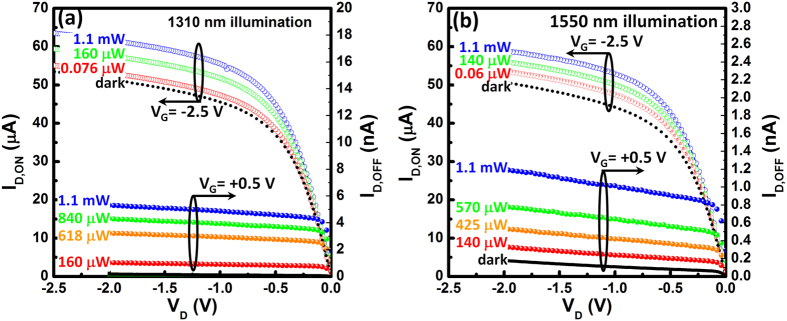
*I*_D_-*V*_D_ characteristics observed for 50 nm Ge-dot *p*-PTs with *t*_ox_ = 3.5 nm under variable-power (**a**) 1310 nm and (**b**) 1550 nm illumination. Distinct current enhancement was achieved for Ge-dot *p*-PTs when electrically-biased in the ON-state (V_G_ = −2.5 V) and OFF-state (V_G_ = +0.5 V). For illumination with wavelength greater than 1100 nm, the photon energy is insufficient to excite electron-hole pairs within Si substrate. The observed photocurrent enhancement indicates tremendous photocarrier generation within the Ge dots.

Another important finding of note is that our Ge-dot PTs also exhibit distinct photocurrent enhancement for both ON- and OFF-states under variable-power 1310 nm and 1550 nm illumination (Fig. 3). For illumination at wavelengths greater than 1100 nm, the photon energy is insufficient to excite electron-hole pairs within Si substrate. Thus, the experimental observation of photocurrent gain for our Ge-dot PTs for 1310 nm and 1550 nm illumination is conclusive proof that the Ge dots are optically active for NIR photodetection.

### Photoresponsivity characterization

**Figure 4:**
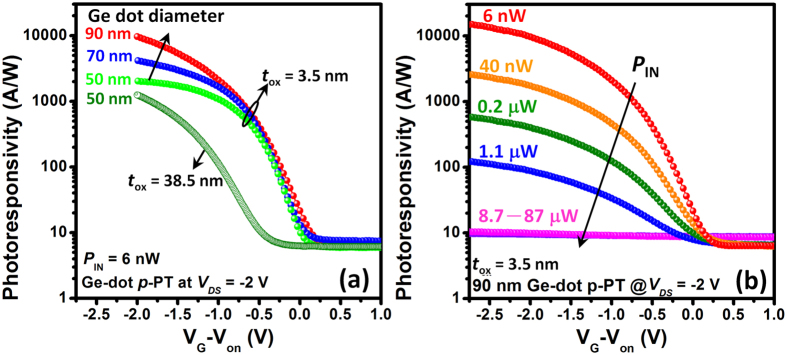
Dependence of photoresponsivity (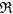) on gate voltage and light power. (**a**) 50–90 nm-diameter Ge-dot *p*-PTs with *t*_ox_ = 3.5 nm and 38.5 nm under 6 nW, 850 nm illumination. The 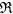 is well modulated by gate voltage and the ON-OFF photoresponsivity ratio is improved by increasing Ge-dot diameter from 50 nm to 90 nm and by decreasing *t*_ox_ from 38.5 nm to 3.5 nm. (**b**) 90 nm Ge-dot *p*-PT with *t*_ox_ = 3.5 nm under variable-power 850 nm illumination from 6 nW–87 μW. 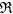
_OFF_ exhibits a superior photocurrent linearity for *P*_IN_ in the range from 6 nW–1 mW, while 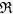
_ON_ decreases with increasing *P*_IN_ and approaches a saturation value of 10 A/W at *P*_IN_ ≥ 8.7 μW.

Photoresponsivity (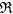), defined as 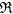 ≡ (***I***_photo_ − ***I***_dark_)/*P*_IN_, is one of the most important figures of merit for a PT since it quantifies the photoelectric gain. Under low-power 850 nm optical pumping, the 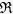 of Ge-dot PTs appears to be well modulated by gate voltage as shown in Fig. 4(a). For example, the photoresponsivity of 50 nm Ge-dot *p*-PT with ***t***_ox_ = 38.5 nm measured at *P*_IN_ = 6 nW, increases from 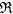
_OFF_ = 6 A/W at *V*_G_ = + 1 V (OFF-state) to 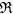
_ON_ = 1.2 × 10^3^ A/W at *V*_G_ = −2 V (ON-state). The ON-OFF photoresponsivity ratio (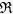
_ON_/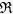
_OFF_) is further improved from 200 to 400 by reducing ***t***_ox_ from 38.5 nm to 3.5 nm due to the improved gate modulation through a thinner gate-dielectric layer. The 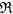
_ON_ of Ge-dot *p*-PT measured at *P*_IN_ = 6 nW also appears to improve with increasing Ge-dot diameter from 50 nm to 90 nm (Fig. 4(a)). Figure 4(b) shows that for the 90 nm-diameter Ge-dot *p*-PT with ***t***_ox_ = 3.5 nm, a very high 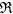
_ON_ of 10^4^ A/W is measured for 6 nW, 850 nm illumination with a corresponding external quantum efficiency of EQE = 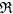
[*h*ν/*e*] = 1.5 × 10^6^%. From the measured values of 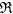, we are able to estimate the specific detectivity D* = 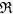
(*A*_Gate_/2*eI*_dark_)^0.5^ at *P*_IN_ > 300 nW, in which the *P*_IN_ regime shot noise is predominant over other noise sources for the Ge MOS structures^15^. At *P*_IN_ = 300 nW, the estimated values of specific detectivity for our Ge-dot PTs being electrically-biased in the ON-state (V_G_ = −2.5 V) and in the OFF-state (V_G_ = +1 V) are 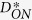 ≈ 1.2 × 10^11^ cm/(W·s^1/2^) and 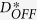 ≈ 3.5 × 10^12^ cm/(W·s^1/2^), respectively. 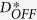 appears to be significantly higher than 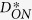 primarily because of an extremely low dark current (I_dark, OFF_ ≈ 4.08 × 10^−11^A versus I_dark, ON_ ≈ 5.95 × 10^−5^A). 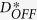 ≈ 3.5 × 10^12^ cm/(W·s^1/2^) for our Ge-dot PTs is comparable to the D* value of 6 × 10^12^ cm/(W·s^1/2^) for state-of-the-art Ge photodetectors measured at 600 nm^15^.

**Figure 5:**
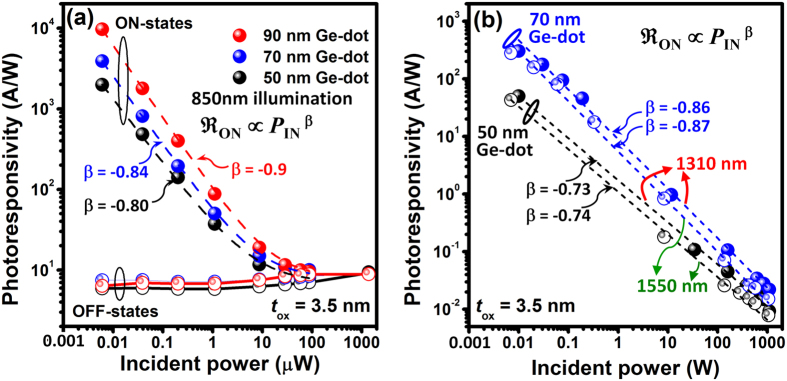
Power-dependent photoresponsivity of 50–90 nm Ge-dot *p*-PTs with *t*_ox_ = 3.5 nm under (**a**) 850 nm and (**b**) 1310 nm, 1550 nm illumination. Our Ge-dot PTs exhibit power dependent 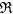
_ON_ with the largest 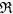
_ON_ obtained for the sub-μW illumination regime for 850 nm–1550 nm wavelengths. The decrease of 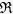
_ON_ with increasing *P*_IN_ can be fitted by a power law of 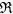
_ON_ ∝ *P*_IN_^β^ with β = −0.7 – −0.9. The dashed lines are fitting curves and the circle symbols are experimental data.

For 850 nm illumination, our 50–90 nm Ge-dot *p*-PTs biased at OFF-state exhibit a superior photocurrent linearity over a wide dynamic range for *P*_IN_ ranging from 6 nW–1 mW with a nearly constant value of 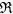
_OFF_ = 6–10 A/W (Fig. 4(b)). While very high 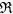
_ON_ is measured for our Ge-dot PTs, 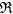
_ON_ appears to have a strong dependence on the excitation power, i.e., the amplification for small optical signals is larger than that for large optical signals. Figures 4(b) and 5(a) show that for 50–90 nm-diameter Ge-dot *p*-PT with ***t***_ox_ = 3.5 nm, 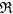
_ON_ decreases with increasing *P*_IN_ and approaches a saturation value of 10 A/W at *P*_IN_ ≥ 10 μW for 850 nm illumination, thereby achieving good photocurrent linearity for *P*_IN_ in the range of 9 μW–1 mW, and possibly for higher illumination powers since 1 mW is the maximum input power available to us given the limitations of our laser sources.

High 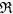
_ON_ of 400 A/W and 300 A/W are also measured for 70 nm Ge-dot PTs at *P*_IN_ = 10 nW, for 1310 nm and 1550 nm illumination, respectively (Fig. 5(b)). These 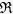
_ON_ values are much higher than the reported values of 42 A/W measured for Si/Ge heterojunction bipolar phototransistors (photoHBTs) at 1 V bias^16^. Our Ge-dot PTs exhibit power dependent photoresponsivity with the largest responsivity obtained for the sub-μW illumination regime for 850 nm–1550 nm illumination (Fig. 5). The measured photoresponsivity decreases with increasing optical power. This phenomenon is typically observed in phototransistors due to high light power affecting the gate and/or base potential^11^,^12^. We found that the decrease of 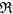
_ON_ with increasing P_IN_ can be fitted by a power law of the form: 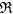
_ON_ ∝ *P*_IN_^β^ with β values between −0.7 – −0.9 (Fig. 5). Also, β appears to decrease with smaller Ge dot size. Similar power law relations have also been observed in PTs based on graphene-MoS_2_ hybrid heterostructures with corresponding β ~ −0.8^17^.

### High Speed Operation

**Figure 6:**
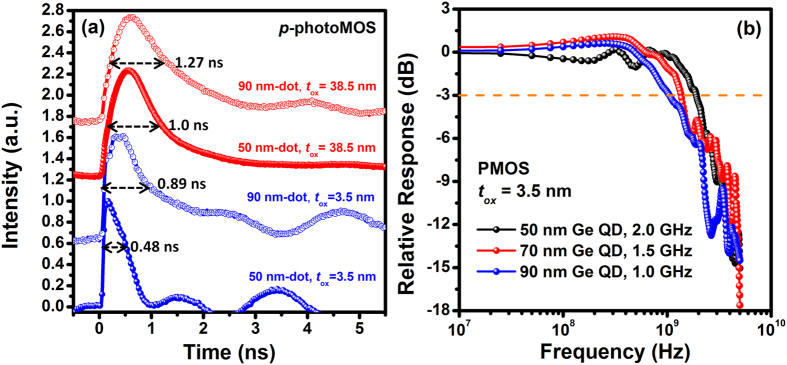
Dynamical photoresponse of the Ge-dot *p*-PTs. (**a**) Normalized temporal response times and (**b**) relative responses to optical excitation as a function of frequency. Decreasing the Ge-dot diameter from 90 nm to 50 nm and reducing *t*_ox_ from 38.5 nm to 3.5 nm significantly improves the temporal response speed and the 3dB frequency bandwidth.

Response time is an important figure of merit for photodetectors. The temporal response time of the Ge-dot PTs appears to be significantly improved by reducing the Ge-dot size from 90 nm to 50 nm as well as by decreasing ***t***_ox_ from 38.5 nm to 3.5 nm (Fig. 6). A response time of 0.48 ns with a corresponding 3 dB frequency of 2.0 GHz is measured for the Ge-dot *p*-PTs with Ge-dot size of 50 nm and ***t***_ox_ = 3.5 nm. The response speed of the Ge-dot PTs is essentially determined both by the lifetime of photo-generated carriers within the Ge dot as well as the gate RC delay that is primarily dominated by the thickness of gate oxide layer. For a given ***t***_ox_, the observation of faster response as measured for smaller Ge-dots, suggests better crystallinity with less defects for the smaller Ge dots.

## Discussion

Our previous report proposed a Ge-dot mediated photoconductive/photovoltatic mechanism to explain the large photocurrent enhancement measured for our Ge-dot PTs biased in both OFF and ON-states^18^. Unlike the conventional Ge-gate^6^,^7^ photoMOSFETs reported to date, in our devices the Ge dot embedded within the gate oxide layer efficiently absorbs light to generate photocarriers, and also effectively confines these photocarriers due to the high potential barrier between the SiO_2_ and the Ge dots. For instance, in the presence of gate voltages that are less than the threshold voltage (|*V*_G_| < |*V*_TH_|) for Ge-dot *p*-PTs biased in the OFF-state, photogenerated electrons confined within the Ge nanodot modulate the vertical *E*-field across the gate oxide layer, thereby reducing the *V*_TH_ that is required to electrically turn on the conducting channel. As a consequence of this modulation, the generation of conductive carriers is promoted leading to photocurrent enhancement. For Ge-dot *p*-PTs biased in the ON-state (|*V*_G_| > |*V*_TH_|), photoelectrons injected from Ge dots into the conducting channel are rapidly swept to the source side by the lateral *E*-field resulting from the *V*_DS_ bias voltage, thereby lowering the potential barrier of the source/channel junction so as to facilitate charge injection from source into the channel. This highly sensitive field effect mediated by the Ge dots is the basis for an efficient intrinsic amplification mechanism that converts the detected photons into large electrical signals (high photocurrents).

**Figure 7:**
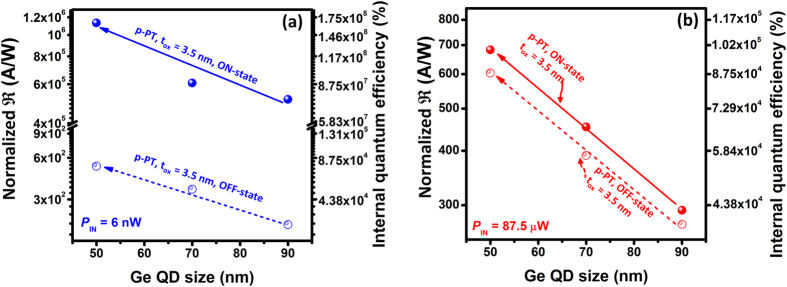
Size-dependent normalized photoresponsivity per unit Ge volume and internal quantum efficiency (IQE) for Ge-dot *p*-PTs with *t*_ox_ = 3.5 nm measured under (**a**) *P*_IN_ = 6 nW and (**b**) *P*_IN_ = 87.5 μW. Decreasing the Ge-dot diameter from 90 nm to 50 nm significantly enhances the normalized photoresponsivity and IQE possibly due to an improvement in crystallinity quality with reduced defect densities.

It is clear to see that for our Ge-dot PTs, the total photocurrent output and 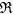 values in all regions of operation can be improved by decreasing ***t***_ox_ and by increasing the Ge-dot size (Figs 4(a) and 5). The process of photocurrent generation consists of photon absorption, photocarrier generation, and photocurrent generation through carrier extraction and transport. A thinner gate-oxide layer is indeed conducive for enhancing gate modulation of the photogenerated carriers within the Ge nanodots because a stronger *E*-field across the gate-stacking heterostructure promotes photocarrier tunneling/injection into the SiGe conducting channel^19^. Also, the channel current of a MOSFET is greatly enhanced by reducing the gate-oxide thickness resulting in increased gate capacitance and improved gate electrostatic control over the channel. For a given ***t***_ox_, one may intuitively rationalize the higher 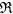
_ON_ measured for larger Ge-dot in sizes as resulting from the greater absorption volumes of the Ge dots. In order to gain further, important insights on the size-dependent, intrinsic efficiency of Ge dots for photoelectric conversion, we have extracted internal quantum efficiency (IQE) values by normalizing 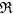 by the ratio of total volume of Ge dots to total product of light absorption length (σ_Ge_) and gate area (i.e., IQE = 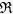/[*V*_Ge dot_/(σ_Ge_ × *A*_Gate_)]) within the gate stacking structures. The results of the normalization are shown in Fig. 7. Remarkably, reducing the Ge dot size from 90 nm to 50 nm leads to an improvement in both normalized 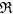
_ON_ and 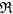
_OFF_ per unit Ge volume by a factor of 2.75 for Ge-dot PTs under both low- and high-power 850 nm illumination conditions. The improved IQE for the smaller Ge dots is possibly due to a significant improvement in crystallinity quality with reduced defect densities and an increased strain^20^, leading to better photoelectric conversion efficiency. As the nanodots get bigger, the statistical probability of having crystalline defects such as dislocations increases. We have also experimentally reported Ge-dot size-dependent compressive/tensile stress generated within the Ge nanodots by the surrounding layers of Si_3_N_4_/SiO_2_in terms of a systematic blue/red-shift in the Raman phonon line as well as a significant enhancement in local lattice deformation as evidenced in transmission electron diffraction patterns. In other words, the smaller the Ge nanodot, the larger the compressive strain. The enhanced strain for smaller Ge nanodots could also contribute to absorption enhancement, resulting in improved photoresponsivity.

Our Ge-dot photoMOSFET has been fabricated in a standard MOS device configuration in which the gate stacking heterostructure of SiO_2_/Ge-dot/SiO_2_/SiGe is simultaneously produced in a single oxidation step. The demonstration of high-photoresponsivity Ge-dot photoMOSFETs for 850–1550 nm photodetection offers great promise for monolithic integration of photodetectors and MOS transistors within standard CMOS processing technology for future Si-based optical interconnect applications. Photoelectric conversion efficiency and response speed are significantly improved by reducing gate-oxide thickness thanks to improved gate modulation and by decreasing Ge-dot size because of the improved crystallinity for small diameter Ge dots. We expect to further improve the photoresponsivity, response time and the 3 dB frequency by increasing the areal density of Ge-dots within the gate stack, since there is still a significant parasitic capacitance produced by the layers of buffer Si_3_N_4_ and the thermal SiO_2_ surrounding the Ge dots.

## Methods

### Self-organized, gate-stacking heterostructure of SiO_2_/Ge-dot/SiO_2_/SiGe-channel formation

The gate-stacking structure is self-organized and formed in a single oxidation step of Si_0.85_Ge_0.15_ nano-pillars patterned over a buffer layer of Si_3_N_4_ on top of the *n*-Si substrate. During the high-temperature oxidation of the poly-SiGe nano-pillars, the Si content in the nano-pillar is preferentially oxidized, squeezing the remaining Ge radially inwards to the centers of the oxidized pillars. Further thermal oxidation results in the consolidation of the Ge nanocrystallites in each pillar via Ostwald Ripening into single spherical Ge dots. These dots also simultaneously migrate through the underlying buffer Si_3_N_4_ layers and achieve contact with the Si substrate. Intriguingly, there is an approximately 3.5 nm-thick amorphous interfacial oxide layer surrounding the Ge dot. This interfacial oxide layer is conformal with the Ge dot and with the Si substrate below. Also, an approximately 20 nm-thick Si_1-*x*_Ge_*x*_ shell is generated within the Si substrate due to the migration of Ge interstitials to the Si substrate from the Ge nanodot. To reiterate, this entire, complex, heterostructure stack occurs within a single oxidation step.

### PhotoMOSFETs fabrication

The fabrication of Ge-dot PTs was initiated using n-Si(100) substrates with resistivity between 0.09–0.7 Ωcm. Following local oxidation isolation processes, BF_2_ (1 × 10^15^ cm^−2^, 40 keV) and phosphorus (1 × 10^15^ cm^−2^, 30 keV) dopants were implanted for the formation of source/drain and substrate electrodes, respectively, for *p*-channel PTs. Next, a tri-layer deposition was conducted using sequential low-pressure chemical vapor deposition of 35 nm-thick Si_3_N_4_, followed by 70 nm-thick poly-Si_0.85_Ge_0.15_, and finally a capping layer of 5 nm-thick SiO_2_. The topmost SiO_2_ layer is deposited as a hard mask for the subsequent plasma etching to define the SiGe nanopillars. The buffer Si_3_N_4_ layer between the Si_0.85_Ge_0.15_ nanopillars and the Si substrate serves as the initial, local source of Si interstitials for promoting the coalescence and migration of Ge nanodots^21^. This thin Si_3_N_4_ layer also acts as an oxidation mask to protect the remaining Si substrate from being oxidized during the subsequent thermal oxidation of the poly-Si_0.85_Ge_0.15_ nano-pillars. The topmost SiO_2_/poly-Si_0.85_Ge_0.15_ layers are then lithographically patterned to create cylindrical poly-Si_0.85_Ge_0.15_ nano-pillars with a pillar density of 10^9^–10^10^ cm^−2^ over the buffer Si_3_N_4_ layers. Next, thermal oxidation at 900 °C in an H_2_O ambient for 28–50 min converts each poly-Si_0.85_Ge_0.15_ nano-pillar (100–210 nm in diameter) to a single spherical, 50–90 nm-diameter Ge dot that is positioned directly beneath each oxidized nano-pillar (Fig. 1). A direct etch-back of the newly-formed SiO_2_ layer over the Ge dots results in two thicknesses of 0 nm and 35 nm for the oxide above the Ge dots, as shown in Fig. 1(a,b), respectively. It is interesting to note that there exists a 3.5 nm-thick interfacial layer of SiO_2_ surrounding the Ge dots. This leads to two gate-oxide thicknesses (***t***_*ox*_) of 3.5 nm and 38.5 nm for the Ge-dot gate stacks. Lastly, a 150 nm-thick indium tin oxide (ITO) layer is deposited and then patterned as a transparent gate electrode with gate-length (*L*_*g*_) of 3 μm and gate-width (*W*) of 70 μm. Subsequently, source/drain metallization and sintering processes are implemented to complete the device fabrication.

### Optical characterization

All characterization was performed under atmospheric ambient, room temperature conditions. Illumination was provided with a lens fiber angled at 80 degrees from the horizontal. Current-voltage (*I-V*) characteristics of Ge-dot PTs were measured using an Agilent B1500A semiconductor parameter analyzer both in darkness and under 850 nm–1550 nm illumination via normal incidence on the transparent ITO electrodes.

### Dynamic photoresponse

The operating frequency response of Ge-dot PTs was characterized using an ultrafast optical pulse laser (850 nm) driven by a pulse generator (to produce impulse modulation with a pulse width of approximately 68 ps) in conjunction with an Agilent 86100 C digital communication analyzer for recording the pulse response obtained from the Ge-dot PTs. The 3 dB bandwidth of Ge-dot PTs was characterized using a continuous-wave 850 nm laser driven by an optical modulator combined with an Anritsu 37397D vector network analyzer for recording the bandwidth.

## Additional Information

**How to cite this article**: Kuo, M.-H. *et al*. High Photoresponsivity Ge-dot PhotoMOSFETs for Low-power Monolithically-Integrated Si Optical Interconnects. *Sci. Rep.* **7**, 44402; doi: 10.1038/srep44402 (2017).

**Publisher's note:** Springer Nature remains neutral with regard to jurisdictional claims in published maps and institutional affiliations.
